# Meeting of the Brazos Valley Medical Association at Calvert, Texas, May 14th and 15th

**Published:** 1901-06

**Authors:** 


### Society Notes.

### Meeting of the Brazos Valley Medical Association at Calvert, Texas, May 14th and 15th.

*Officially reported for the Texas Medical Journal.*

The Brazos Valley Medical Association met at 10:30 a. m., May 14th. President Pro Tem. Dr. Daniel Parker, of Calvert, in the chair.

Invocation by Rev. C. J. Oxley.

The address of welcome on the part of the citizens was delivered by Mayor C. S. Meredith.

Dr. Daniel Parker made the address of welcome on part of the medical profession. Response by Dr. M. K. Lott, of Cameron.

Reading of the minutes of the previous meeting by the Secretary. Minutes adopted, and Association adjourned until 2:30 p. m.

Meeting called to order at 2:30 p. m.

The Judicial Committee reported for membership the following: Drs. F. E. Daniel, of Austin, editor TEXAS MEDICAL JOURNAL ("Red Back"); Allen J. Smith, University of Medicine, at Galveston; E. Wilder, Calvert; A. L. Mondrick, Bryan; Howard Gilmore, Hayes; J. L. Cass, Cameron; W. J. Fountaine, Jones' Prairie, and D. H. West, Calvert. All elected to membership.

President J. P. Oliver, of Caldwell, spoke as follows:

"*Gentlemen of the Brazos Valley Medical Association:*

"Permit me to congratulate you upon the successful organization of your eleventh semi-annual meeting. So far as my knowledge extends, your organization has broken the record and surpassed any other similar medical body. In your mutual work not a harsh word has been spoken even in heated debate against a brother member, which should be commended and perpetuated throughout all subsequent meetings. I sincerely trust that in leaving your homes and loved ones, to meet in this beautiful city, you have come in the interest of our beloved profession and science. There are some points which might engage your thoughts and a portion of your time profitably. A permanent home for the Association to meet at every few years; the enterprise of building up at this point a good medical library under such rules as the Association should suggest which might have for its object the formation of a great institution for the treatment of the sick and the afflicted, under the auspices of the Brazos Valley Medical Association; to instruct the Secretary to make effort to collect past dues by drawing upon delinquents through the banks if necessary, and in case of refusal to pay, to notify the Association and drop such names from the roll; to increase the fees for membership to one dollar, and to make annual dues one dollar; to increase salary of Secretary."

On motion, the Secretary was instructed to appoint one member from each county represented to act as a committee and to report on the foregoing recommendations. The following physicians were named: Drs. H. W. Cummings, Robertson; W. C. Blalock, Limestone; R. E. B. Bledsoe, Burleson; C. M. McLarty, Lee; J. E. Thompson, Galveston; Joseph Mullen, Harris; M. K. Lott, Milam; S. P. Rice, Falls; J. A. T. Page, Freestone; M. L. Langford, Navarro; A. G. Barnhill, Goliad; R. W. Nobles, Bell; W. G. Peterson, Calhoun; E. O. Boggs, Leon; J. D. Porter, Williamson; D. L. Peeples, Grimes; F. E. Daniel, Travis.

The committee reported as follows:

"We, your committee appointed to consider the recommendations of the President, beg leave to report as follows:

"It is not our opinion that it is a wise policy to locate this Association in any particular city, as the moving of same adds such life as would be lost by locating. We further believe that the plan of organizing a library and beneficiary fund is impracticable, especially at this time. We are unanimously in favor of increasing the salary of the Secretary $25.00 per year; to raise the membership fee to one dollar and the annual dues to one dollar. We desire to make in this connection the following recommendation: That while we most heartily enjoy the usual banquet given us by the local fraternity, yet we believe that it is usually so much a task, and in some instances is an impediment to the smaller towns in our district, undertaking to entertain the Association, we would express it as our opinion that such entertainments should be discontinued as a rule, and while we are of such opinion we will be highly pleased to accept any entertainment offered us.

"Respectfully submitted,

"W. C. Blalock,
"R. E. B. Bledsoe.
"M. K. Lott,
"Geo. R. Tabor,
"H. W. Cummings,
"For Committee."

The report of committee was unanimously adopted (although a fair number—including the Secretary—turned pale at the bare possibility of "no banquet" at the close of any semi-annual meeting of the Association).

It was the sense of the Association that the Secretary make earnest effort to collect arrearages in dues and in case of failure to report names to be placed on suspended list. The Secretary was instructed to secure services of a stenographer for each semi-annual meeting.

Dr. Lott stated, par parenthesis, that all the Association now needed was a good membership, which we have, prompt payment of dues and the society would be amply self-sustaining.

SECTION ON MATERIA MEDICA.

Report of Chairman, Dr. R. E. B. Bledsoe, was received.

Paper, "The Drug Habit—Morphine, Cocaine, Chloral and Whiskey—Treatment and Cure," by Dr. M. K. Lott, Cameron. Uses as treatment hydrobromate hyosciene. After forty-eight hours gives pilocarpine one-eighth grain and strychnine one-twentieth grain, once or twice daily. For diarrhea gives one-half drachm bismuth subgallate and F. E. Coto bark thirty to forty drops, as may be needed. Then nourishing food. For sleeplessness advises cold bath. The antidote for whiskey is likewise the antidote for chloral, cocaine and morphine.

A rising vote of thanks was tendered Dr. Lott for his very able paper.

Paper, "The Treatment of Pneumonia with Special Reference to Carbonate of Creosote," by Dr. H. W. Cummings, Hearne. Has treated twenty cases this year and without loss.

Dr. S. P. Rice, of Marlin, endorses the treatment and by its aid has cured a large number of cases.

President Oliver requested Dr. Daniel to address the Association on the subject of smallpox, and to include quarantine.

Dr. Daniel complied, giving some interesting facts relating to quarantine and smallpox. He spoke of smallpox in the German and French armies during Franco-Prussian War, 1870-71. When vaccination was introduced in 1874 the dread disease was virtually stamped out. He favors compulsory vaccination when compulsion is necessary to public safety.

Dr. Tabor followed. Has seen about one thousand cases, as health officer in Brazoria county. Disease generally mild. Negroes have a horror of vaccination.

Dr. N. Cass, health officer of Milam county gives his treatment of smallpox. If he can commence treatment within six days after the eruption has taken place, he has no fear of the result. If called to a case in a family, he at once commences treatment; does not isolate the case; permits patient to sleep as usual and eat at table with other members of the family, but the disease does not spread. He advises sponging the body well, especially the head, twice daily, with a solution of the bi-chloride of mercury—one ounce dissolved in one-half gallon of water and used warm. In two or three days he reduces the mercury to one ounce to one gallon of water, bathing as before for two or three days, when he again reduces the quantity of mercury to one-half ounce to one gallon water. He concludes his treatment by bathing patient twice daily in a bath of hot salt and water until scales disappear. Has lost no cases under this treatment.

Paper, "Apomorphia, as Applied in Asphyxia, Drowning, Lightning Stroke, Emetic Ejecting Poisons from the Stomach, Labor, Fever, Strangulated Hernia, Etc.—Its Origin and Therapeutics," by Thos. J. Pugh, M. D., Bryan, Texas.

He has used this agent in profound asphyxia, drowning, strangulated hernia, morphine poisoning, etc., and always with success. Gives one-tenth grain hypodermically and repeats dose, if necessary.

Dr. Abney has not used apomorphia as Dr. Pugh has done, but has made extensive use of it, by mouth, as an expectorant.

President Oliver suggests this remedy in delirium tremens, one-tenth grain hypodermically.

Dr. Carroll keeps apomorphia in well-stoppered bottles. He states: If a person has never opened his mouth or eyes, he will learn how in a few moments after an injection of this substance. Has given it in cases of hysterics and always with rapid recovery. In sour stomach, colic, etc., gives one-tenth grain; relief rapid. Apomorphia is rapidly eliminated.

### SECTION ON GENERAL MEDICINE.

Report of Chairman, Dr. J. H. Jenkins, Caldwell.

Paper, "The Systematic Doctor," Dr. R. E. Carroll, Calvert. He advises a record of every case. Does not believe in cutting down original charge. Attend strictly to business. Make out monthly statements, but in no case allow a statement to pass beyond a quarter. Do not place a patient's name on your books if the pay is doubtful. Do not fail to keep correct record of temperature. He advocates a card system which he explains.

Paper, "Typho-Malarial Fever," Dr. E. H. Gray, Milano. Duration from fourteen to twenty-five days. He has never lost a case. Gives mercury, acetanilid and quinine to control fever, but if no success attends he uses turpentine emulsion.

Dr. Bledsoe is sometimes called to see cases of typhoid fever. One case, a little girl, fever, hemorrhage, etc. Gave quinine; recovery. For an adult he has given sixty grains quinine one day, eighty grains the next and the next from one hundred and ten to one hundred and twenty grains, ending in recovery.

Dr. Moses does not believe it is "typho-malarial," but thinks it is either typhoid or malarial, and favors large doses of quinine.

Dr. Nicks says we must revise the text-books or alter this disease.

Dr. Collard says those who have discussed this paper are all right and all wrong. The fever is neither typhoid nor is it malarial, but for want of a better name we call it "slow fever." He also stated when Peyers' patches are inflamed it is typhoid. He has performed autopsies which verified this diagnosis.

The secretary objects to killing a man to find out what is the matter with him.

Meeting adjourned to 8:30 a. m., May 15th.

SECOND DAY—MORNING SESSION.

Meeting called to order at 8:30 a. m. by President Oliver.

Paper, "Malarial Hematuria, with Thoughts on General Medicine," by Dr. J. M. Nicks, Stone City. Symptoms as usual in incipiency, and ending generally with health or syncope. States when plasmodium malaria is found, quinine is the remedy. The bile is more poisonous that the urine. He has caused the urine to return to the red color by giving sulph. quinine after the red color had disappeared. In these cases he uses the bi-muriate of quin. and urea, hypodermically. Advocates turpentine and sweet spirits of nitre; also uses tinct. mur. iron. Makes free use of calomel in this disorder, so often fatal.

Dr. Gray had only seen a few cases. Believes in quinine, hypodermically, in ten-grain doses.

Dr. Collard does not think the kidneys primarily diseased, but that they are called upon to carry off the debris, and by overwork become diseased. He had seen a case who rode several miles to see him, not thinking he had hematuria, but who voided in his presence the characteristic urine. In the intermittent type, quinine is indicated.

Dr. Bledsoe thinks in malarial cases there is real hematuria, and in the intermittent form it is hemoglobinuria.

Dr. Rice believes the pressure on the kidneys is what prostrates and ruins them.

Dr. McKnight thinks this pressure is caused by the great effort of the kidneys in unloading the blood poison. Does not favor quinine.

Dr. Jenkins objects to quinine, as does Dr. Gilmore. Dr. Jenkins has brought back bloody urine twice in the same case—a little girl—by use of quinine.

Dr. Briggs (Secretary) advocates bi-chlor. mercury, calomel, strychnia, arsenic, sanmetto, acetate potassa in solution, and sweet spirit nitre. Does not believe in the use of quinine in any form, but if urgently insisted upon would prefer the bi-muriate quinine and urea, hypodermically.

Under the head of Surgery, we have paper: "The Incision in Appendicitis," by Dr. J. E. Thompson, of Medical Department University of Texas, at Galveston. He advises the rectus incision as being the safest and easiest. Retracts the nerves with blunt retractors, and uses great care not to injure the deep iliac artery. Dr. Thompson brings to his aid, for the purpose of explanation, two beautiful colored plates, made by him, which greatly simplifies the operation.

Dr. Blalock thinks the rectus incision the best and safest. Has performed the operation several times. He also likes McBurney's operation.

Dr. Collard states what was known as bilious colic, etc., is a thing of the past, and that everything of that nature is now known and diagnosed as appendicitis.

Dr. Thompson, by request, gave advice regarding abdominal troubles. He thinks the country practitioner should not refuse an abdominal operation because he has not good surroundings, but should say, "I will do the best I can." He should use an exceedingly sharp knife, and ligatures should be of catgut and silk worm. Two or three pair of blunt retractors should also be at hand.

A rising vote of thanks was tendered Dr. Thompson for his most excellent paper, and for his earnest address.

Dr. Lott, chairman of Section on Obstetrics and Pediatrics, by an oversight had left his report at home, but promised to send the same to the Secretary for his disposal.

Paper, "Post Partum Hemorrhage," by Dr. B. S. Ezell, of Kosse. States symptoms are so plain that every physician can readily read it. Uses one-twentieth grain strychnia in every case. Introduces iodoform gauze and packs vagina.

Dr. Garrett elevated hips. Objects to tamponing uterus. Compresses womb and has an assistant to draw points of fingers together and make pressure over it. Gives hypodermics of ergot. He often sponges out uterus with vinegar. Uses strychnia and digitalis to support heart. In cases of emergency, he uses sponge saturated with turpentine, in absence of vinegar, and thus controls the hemorrhage.

Dr. Blalock uses strychnia freely and makes pressure over uterus with the cupped hand for twenty or thirty minutes. Also uses hypodermics of ergot.

Dr. Torbett cites a case in which he used many remedies, but failed. He, after using strychnia, introduced ice, gave ergotole, etc., and saved patient. He learned that this same lady two years afterwards gave birth to a child, had post partum hemorrhage and died.

Dr. Carroll advises the normal hot salt solution.

Dr. Gilson tests the pulse and believes that an indication of the coming storm.

Paper, "Criminal Abortion," by Dr. J. P. Oliver, of Caldwell. Speaks of instrumental and other means used to produce this criminal offense. In 1871 laws were passed in all but three States making abortion a criminal offense. Laws of Pennsylvania and Texas are about the same. For producing abortion, selling instruments or medicines for same, which is made a felony, is punishable by penal servitude for three years.

Dr. Thompson endorses the paper.

Dr. Blalock reports a case of complete inversion of uterus immediately following delivery.

This closed the work on sections.

The election of officers resulted as follows:

Dr. Daniel Parker, of Calvert, President.

Dr. F. R. Collard, of Wheelock, First Vice-President.

Dr. S. P. Rice, of Marlin, Second Vice-President.

Dr. J. W. Hudson, of Milano, Treasurer.

Dr. W. B. Briggs, of Easterly, Secretary.

Drs. H. W. Cummings, Hearne; G. M. Abney, Franklin; M. K. Lott, Cameron; Geo. R. Tabor, Bryan; J. M. Nicks, Stone City, Judicial Council.

All the officers were elected by acclamation.

Dr. J. P. Oliver made his retiring address as President of the Brazos Valley Medical Association.

President Parker being unavoidably absent, Dr. F. R. Collard was escorted to the chair and made the address in behalf of President-elect Dr. Parker.

The Secretary being called upon, thanked the members for the honor conferred upon him by his election for the fifth time to the office of Secretary.

Bryan was selected as the next place of meeting of the Association, the second Tuesday and Wednesday in November.

The following resolution was adopted:

Resolved, That the members of the Brazos Valley Medical Association, individually and collectively, extend to the good citizens of Calvert, and especially to the ladies, their high appreciation and gratitude for the princely manner in which they have been entertained and their every wish supplied. We will ever cherish a happy memory of our stay in their midst.

S. P. Rice,
L. M. Bassett,
M. K. Lott,
Committee.

Adjourned to meet at Casimer's Opera House, at 8 p. m., to hear address of Dr. Allen J. Smith, of Medical Department of the University of Texas, at Galveston. Subject: "Mutual Relation of Faith and Science." This was the grandest lecture ever delivered in this section, and must be heard to be fully appreciated. It was a treat never to be forgotten.

After the lecture came the banquet at the city hall. Here the tables were loaded with everything appetizing. Toasts were called for and responded to. About the hour of midnight the physicians took their departure homeward, but they will never forget Calvert—never.

W. B Briggs,
Secretary.

---

Binz Bldg., Houston, Texas, May 10, 1901.

Dear Doctor: The South Texas Medical Association will hold its next meeting in Houston, June 20th and 21st. It has no medical board to select nor political axe to grind, and the eye, ear, nose and throat men will not be frozen out.

Please come and give us something of your own selection which will be of interest relative to eye, ear, nose or throat, either in report of cases or a thesis pertaining to either, and we promise you a cordial welcome and a respectful hearing. Write us as soon as possible the title of your paper.

Respectfully,
DR. E. P. DAVISS,
Chairman Sec. Eye, Ear, Nose and Throat,
South Texas Medical Association.

---

### South Texas Medical Association.

